# Myeloid Cell Interaction with HIV: A Complex Relationship

**DOI:** 10.3389/fimmu.2017.01698

**Published:** 2017-11-30

**Authors:** Vasco Rodrigues, Nicolas Ruffin, Mabel San-Roman, Philippe Benaroch

**Affiliations:** ^1^Institut Curie, PSL Research University, INSERM U932, Paris, France; ^2^Institut Curie, PSL Research University, UMR3216, Paris, France

**Keywords:** macrophages, sensing, viral assembly, antiretroviral therapy, reservoir, virus-containing compartment, restriction factors

## Abstract

Cells of the myeloid lineage, particularly macrophages, serve as primary hosts for HIV *in vivo*, along with CD4 T lymphocytes. Macrophages are present in virtually every tissue of the organism, including locations with negligible T cell colonization, such as the brain, where HIV-mediated inflammation may lead to pathological sequelae. Moreover, infected macrophages are present in multiple other tissues. Recent evidence obtained in humanized mice and macaque models highlighted the capacity of macrophages to sustain HIV replication *in vivo* in the absence of T cells. Combined with the known resistance of the macrophage to the cytopathic effects of HIV infection, such data bring a renewed interest in this cell type both as a vehicle for viral spread as well as a viral reservoir. While our understanding of key processes of HIV infection of macrophages is far from complete, recent years have nevertheless brought important insight into the uniqueness of the macrophage infection. Productive infection of macrophages by HIV can occur by different routes including from phagocytosis of infected T cells. In macrophages, HIV assembles and buds into a peculiar plasma membrane-connected compartment that preexists to the infection. While the function of such compartment remains elusive, it supposedly allows for the persistence of infectious viral particles over extended periods of time and may play a role on viral transmission. As cells of the innate immune system, macrophages have the capacity to detect and respond to viral components. Recent data suggest that such sensing may occur at multiple steps of the viral cycle and impact subsequent viral spread. We aim to provide an overview of the HIV–macrophage interaction along the multiple stages of the viral life cycle, extending when pertinent such observations to additional myeloid cell types such as dendritic cells or blood monocytes.

## Introduction

The introduction of antiretroviral therapy (ART) to treat HIV infection in the mid 1990s was met with extraordinary success and dramatically improved the lives of patients, by turning a deadly infection into a manageable chronic disease. However, while able to prevent progression to AIDS, ART cannot eradicate HIV from the body, and a viral reservoir quickly rebounds after interruption of the therapy. In addition, HIV patients under suppressive therapy are at elevated risk of developing several non-AIDS related diseases, including cognitive impairment and cardiovascular problems.

HIV mainly replicates in CD4 T cells and macrophages in the body. Loss of CD4 T cells has long been known as the major pathological event leading to AIDS. In macrophages, HIV infection does not induce immediate cell death and viral replication proceeds for extended periods of time.

Macrophages maintain tissue homeostasis by performing crucial housekeeping tasks. Their ubiquitous distribution in the body allows HIV to disseminate into organs and tissues and establish compartmentalized infection. Macrophages are also an important effector arm of the innate immune system. These cells detect HIV infection and express cellular factors that severely restrain the capacity of the virus to replicate.

Here, we discuss the interplay between HIV and macrophages. We review recent work highlighting the unique interaction between HIV and macrophages, at the cellular level. We further discuss evidence pointing to a role for macrophages as cellular reservoirs of HIV during ART and how they participate in the pathological morbidities that prevail in patients under therapy.

## Macrophage Ontogeny and Function

Macrophages populate virtually all tissues of the body, where they perform a multitude of functions that are essential for tissue homeostasis, architecture, and protection (1). This wide range of macrophage action was described more than a century ago by Elie Metchnikoff. In his pioneering work, Metchnikoff observed the swarming and subsequent clearance of foreign objects by phagocytic cells in starfish larvae and water fleas (1). He correctly foresaw the importance of macrophages in the removal of obsolete cells, pathogen elimination, or sterile inflammation (2, 3).

Tissue macrophages have classically been considered as originating exclusively and in a continuous manner from bone marrow-derived monocytes, as part of the mononuclear–phagocyte system, a concept put forward by Van Furth in the 1970s (4). However, fate-mapping studies over the past decade have drastically changed our views on macrophage ontogeny. It is now widely accepted that many tissues are seeded with macrophages derived from the yolk sac or the fetal liver, during embryonic development [reviewed in Ref. (5)]. Once at their site of residency, macrophages proliferate locally to maintain a population size able to meet the requirements of the developing tissue or organ (6). The ability to self-renew suggests the existence of a subpopulation of tissue-resident macrophages with stem cell properties and capable of asymmetric cell division, but no such cell has yet been described in the tissues (6), with the possible exception of a subpopulation of epidermal Langerhans cells (7). Alternatively, the whole population of macrophages residing in a given tissue may be endowed with self-renewal potential, as suggested in studies with microglial cells or peritoneal macrophages (8–10). In some tissues, such as the brain or the liver, the resident macrophage population appears to be exclusively derived from embryonic cells throughout all adulthood (6). While monocytes may infiltrate these tissues under inflammatory or pathologic conditions, and differentiate into macrophages, they do not become part of the stable resident population (11). In stark contrast, embryonic macrophages that seed the gut prenatally appear to be completely replaced by monocyte-derived cells after birth (12). The factors that dictate this differential capacity of embryonically or monocyte-derived cells to stably engraft different tissues are not well understood and are an area of active research (6).

In their tissues of residency, macrophages perform a wide range of tasks. Some of these functions, such as apoptotic cell removal or extracellular matrix (MA) remodeling, are required in all tissues to different extents, indicating that macrophages are engaged in cross talks with their local microenvironment. The capacity to perform such general functions appears to be imprinted in the whole macrophage lineage and possibly involves the role of master transcription regulators such as PU (13). Other functions, by contrast, are specific to certain tissues. For instance, alveolar macrophages are specialized in clearing excessive surfactant, while macrophages of the red pulp of the spleen recycle iron from senescent erythrocytes (6). These site-specific functions are presumably imprinted on macrophages by tissue-specific signals and will induce transcriptional programs that define macrophage populations in different tissues (13).

Tissue macrophages are further subjected to environmental cues that occur in non-homeostatic conditions such as inflammation. Evolution has shaped macrophages as primary tissue sentinels (14). These cells are equipped with a broad range of receptors capable of detecting molecular patterns from all classes of microbes and multiple types of tissue damage, as well as receptors for chemokines and cytokines produced by immune cells (1). Integration of these multiple signals leads to what is commonly known as macrophage polarization (15). For instance, in an infected/inflamed tissue, macrophages may encounter microbial products such as LPS or be exposed to T cell-derived IFN-γ, leading to a polarized state known as M1, that is highly efficient in killing intracellular or ingested pathogens (15). Importantly, the majority of the knowledge gathered on macrophage polarization derives from well-defined *in vitro* experiments (16). These studies led to the M1 versus M2 model of macrophage polarization, which is unlikely to capture the complexity and the diversity of signals that macrophages can integrate *in vivo*. Furthermore, these polarizing stimuli act upon macrophages with previously imprinted tissue-specific programs. As such, similar polarizing signals probably lead to distinct phenotypes in macrophages from different tissues (13).

A wealth of information on the ontogeny, differentiation, and function of macrophages, derived from multiple studies in recent years, has profound implications in how we perceive the role of the macrophage in pathological settings, such as cancer, metabolic disease, or infections like HIV.

## Macrophages During Acute and Chronic HIV Infection

The first description that tissue macrophages were permissive to HIV infection and capable of replicating the virus came in 1986, from the lab of Robert Gallo (17), amid the fast-paced period that characterized the early years of HIV/AIDS research. That very same pioneering study further provided the initial evidence that macrophages produce HIV for extended periods of time, hence coping with viral-induced cytopathy (17). In addition, the study revealed that in macrophages, HIV accumulates in apparent intracellular compartments absent from T cells.

HIV can infect macrophages as these cells express both the viral entry receptor, CD4, and co-receptors, CCR5 and CXCR4, that bind the viral envelop protein, gp120. Macrophage infection by HIV requires initial adsorption of the virus to the cell surface, mediated by lectin-like receptors, integrins, and heparan sulfate proteoglycans (18). Entry then probably takes place following virion internalization into macropinosomes (19) or endosomes, where fusion between the viral envelope and the host cell appears to occur (20), as recently proposed by a study following the internalization of fluorescent quantum dots encapsulated by infectious HIV-1 particles in primary macrophages (21).

Classically, macrophage-tropic viruses (M-tropic) were thought to exclusively employ CCR5 for entry (R5 viruses), while CXCR4-using strains (X4 viruses) were viewed as unable to enter macrophages and establish productive infection (22). This simplified categorization of macrophage tropism based on co-receptor usage has been proven imperfect as many R5 viruses are unable to infect macrophages (23), whereas some X4 isolates can (24). While co-receptor usage may frequently predict macrophage tropism, categorizing a virus as M-tropic requires demonstration of its ability to replicate *in vitro* in macrophages, although, understandably, this may not be a practical approach to test every isolate (18).

Transmitted/founder (T/F) viruses are the viral variants that initiate infection in a new host, at genital or rectal mucosal surfaces. Their sequences can be inferred by the mathematical modeling of virus evolution after single-genome amplification analysis of the plasma viral population (25). These types of analyses, across multiple studies, support the idea that most infections are initiated by a single or a very limited number of founder viruses (26). Biological characterization of T/F viruses demonstrated that they are usually unable to replicate in macrophages (27, 28), possibly due to the lower densities of the CD4 molecule on the macrophage surface, as compared with CD4 T cells (29). This suggests that macrophages are not an important source of viral replication in the initial stages of infection, emerging only later, as the virus adapts to infect cells with a lower CD4 density at the surface. In agreement, studies with mucosal explants from the human reproductive tract (30–32), or in non-human primates (33) support the idea that CD4^+^ T cells are the crucial targets at very early time points of infection.

Following migration from mucosal entry points into regional lymph nodes, *via* yet poorly described mechanisms, HIV rapidly disseminates systemically in the host. In SIV-infected rhesus macaques, viral spread to distal tissues such as the gastrointestinal (GI) tract or the spleen can be detected as early as 1 day after intravaginal inoculation and systemic distribution of SIV was observed by day 7 (34). This rapid but clinically silent spread is followed by the acute phase of HIV infection characterized by unrestrained viral replication in multiple tissues (35–37).

Macrophages are likely targets of HIV during the acute phase of infection, as viral nucleic acids have been detected in tissue macrophages from multiple organs in infected patients. These include Kupffer cells in the liver (38), microglial cells in the brain (39), alveolar macrophages in the lung (40), and intestinal macrophages obtained from several segments of the GI tract (41, 42). Importantly, replication-competent virus can be recovered from cultures of macrophages purified from lymphoid tissues of acutely infected rhesus macaques (43), implying that productive infection is taking place. It remains unclear how HIV disseminates to establish infection in these cells and tissues.

Monocytes can seed many tissues and differentiate locally into macrophages, turning this cell type into a potential vehicle for HIV dissemination across the myeloid compartment. Several reports claim indeed that replication of HIV-1 can take place *in vivo* in monocytes, even in patients under ART (44–46). Infected monocytes have been proposed to play a key role in viral dissemination to the brain due to their capacity to cross the blood–brain barrier (47), see Ref. (48).

At their sites of residency, macrophages constitutively patrol the tissues for danger signals, while also performing several housekeeping tasks. Interestingly, through the action of the viral accessory protein Nef, HIV is capable of reprogramming the migration of macrophages and selectively promotes a mesenchymal type of migration, while inhibiting the amoeboid type (49). The mesenchymal mode of migration is characterized by extensive extracellular MA remodeling, thus allowing the invasion of dense microenvironments, which may further promote viral dissemination and persistence. The relevance of these findings is supported by the increased accumulation of macrophages in the tissues of mice engineered to express the HIV Nef protein (49).

Alternatively, migratory, infected CD4^+^ T cells may serve as vehicles for HIV systemic dissemination, as suggested by intravital microscopy in infected humanized mice (50), and possibly transmit the virus to tissue-resident macrophages. Interestingly, during the acute phase, SIV-DNA-positive myeloid cells present in lymphoid tissues also contain rearranged T cell receptor DNA (51). This suggests that phagocytosis of infected T cells allows macrophages to acquire viral DNA, which is presumably taken to the macrophage degradative compartments for destruction preventing potential infection of macrophages (51). However, at least *in vitro*, cultured macrophages become productively infected after ingesting infected T cells (52). This mode of direct T cell-to-macrophage HIV transmission results in more efficient macrophage infection than exposure to cell-free virus (52). While this mechanism has yet to be demonstrated *in vivo*, it seemingly represents a strategy employed by HIV to maximize its spread, by exploiting the extensive phagocytic capacity of macrophages (53).

Chronic untreated HIV infection leads to extensive depletion of the body’s CD4^+^ T cell pool and progression to AIDS (54). Concurrently, the viral population evolves to become more M-tropic (55), presumably because extensive CD4^+^ T cell loss makes the macrophage the most abundant cell target in advanced disease. Rhesus macaques treated with an antibody depleting CD4^+^ T cells before SIV infection mimic this advanced stage AIDS (56). In these animals, macrophages represent about 80% of the SIV-RNA^+^ cells in the tissues with evidence of productive infection of macrophages from lymphoid tissues and the brain, frequently associated with activation markers. Remarkably, plasma viral loads were two logs higher in depleted animals as compared with CD4^+^ T cell-sufficient controls, which led to rapid disease progression (56). Thus, in the context of CD4^+^ T cell depletion that might reflect the advanced AIDS status, extensive activation and viral replication in macrophages drives a precipitous progression of clinical disease.

## Macrophage Sensing of HIV and Intrinsic Restrictions to Viral Replication

The macrophage paradox refers to the fact that macrophages represent both the first line of defense against many pathogens, including viruses, and yet are exploited by many of these pathogens as their favorite cellular niche for replication (57). Such is the case of HIV-1, which efficiently replicates in macrophages. However, as sentinel cells, macrophages are equipped with a range of sensors that detect ongoing infection at many steps of the viral cycle and trigger cellular responses that will activate antiviral immunity (58). A number of these induced antiviral effector genes, known as restrictions factors, will block infection at specific steps of the viral life cycle (59). Thus, from the viral perspective, the extent to which HIV-1 replicates in macrophages must be tightly regulated as to ensure viral transmission/dissemination, while avoiding significant antiviral responses. Such delicate balance is achieved by a combination of precise employment of viral accessory proteins and usurpation of the normal function of cellular factors. Complete reviews devoted to the various restriction factors are available (59), we will focus here on factors that play important roles in infected macrophages.

Studies examining the initial stages of the viral cycle, i.e., upon viral entry and retro-transcription (RT) of the viral RNA into cDNA in the cytosol, proposed that HIV-1 escapes early innate sensing in myeloid cells before viral DNA integration. This would result from a combination of shielding the newly synthesized cDNA by viral and cellular factors (60, 61), and maintenance of very low levels of cytosolic viral cDNA due to the action of the cellular nuclease TREX1 (62, 63). However, induction of a weak, yet detectable, interferon-stimulated gene (ISG) response after HIV-1 infection of macrophages has also been reported (64, 65), with type I IFNs levels remaining undetectable (64, 66).

**Figure 1:**
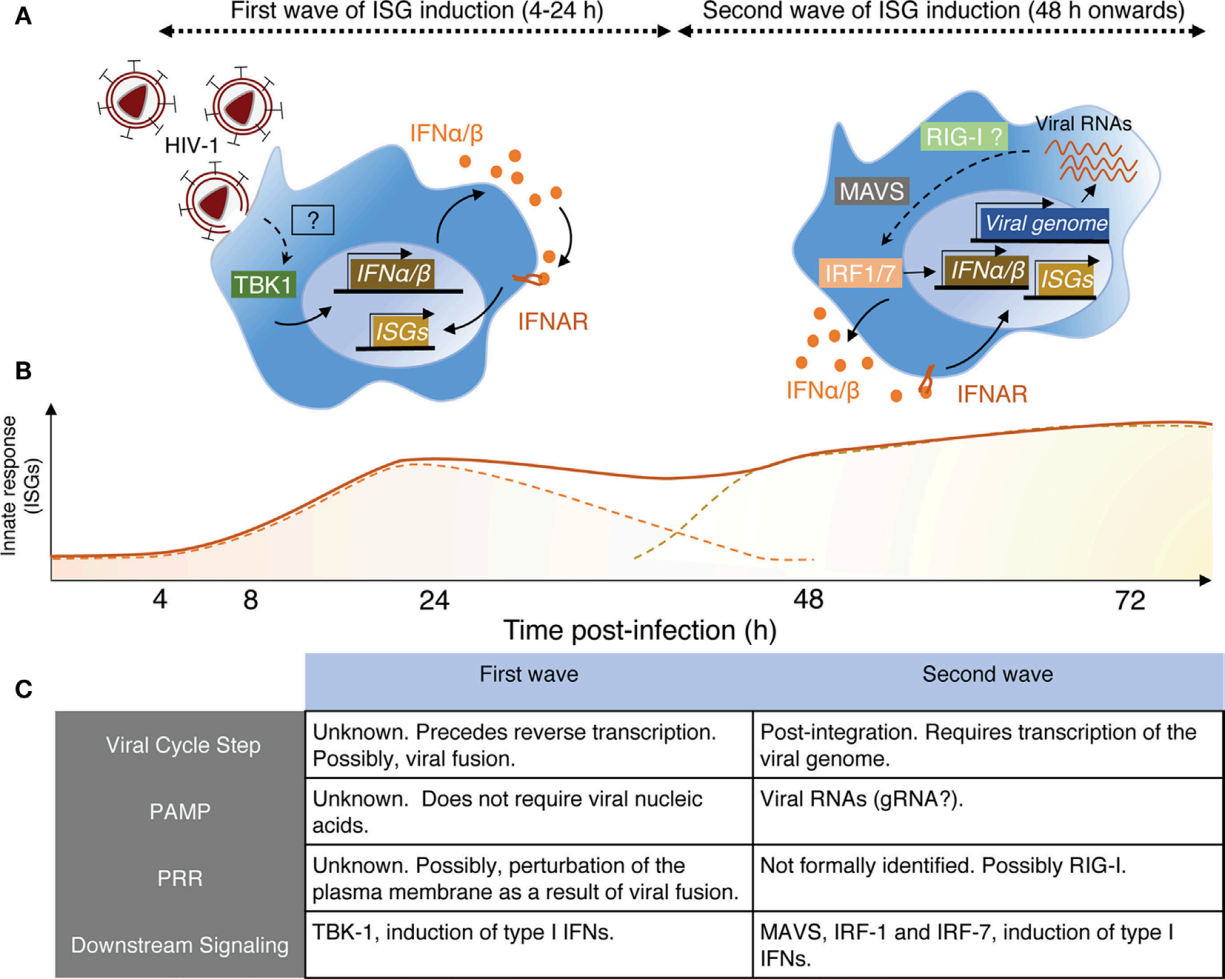
Schematic view of HIV-1 sensing by macrophages. **(A)** Macrophages sense HIV-1 at two independent steps of the viral cycle. *Left panel*—Early sensing of HIV-1 by macrophages requires viral fusion with the plasma membrane but precedes retro-transcription (RT). This sensing step is detectable by 4 h after cell exposure to the virus and declines after 24 h when RT is inhibited. While the actual sensor involved remains to be identified, it activates the kinase tank-binding kinase-1 (TBK1), leading to production of type I IFN, signaling *via* IFNAR, and triggering of interferon-stimulated genes (ISGs). *Right panel*—The second wave on HIV-1 sensing is measurable only 48 h after cell exposure to the virus. It requires integration of the viral genome in the host DNA and transcription of viral RNAs, which appear to be the viral component triggering the late ISG response. Here also, the actual sensor remains to be identified, but retinoic acid-inducible gene I (RIG-I) is a likely candidate as the signaling cascade involves the adaptor MAVS and IRF-1 and IRF-7, leading to type I IFN production. **(B)** Schematic representation of the two ISG waves induced by the sensing steps described in panel **(A)**. The full line represents the putative measurable ISG response, whereas the dashed lines indicate the contribution of the individual waves for the measurable response. **(C)** This table resumes the main characteristics associated with the two sensing mechanisms through which macrophages detect HIV-1 and was established based on Ref. (67, 70).

Examining these apparent discrepancies, we recently confirmed this transient response of monocyte-derived macrophages (MDMs) to HIV-1 infection, detectable as soon as 6 h postexposure and peaking at 24 h (67). Such response induces an ISG signature and depends on the induction of low levels of type I IFN. This sensing step is macrophage specific as it does not occur in monocyte-derived dendritic cells (MDDCs) exposed to HIV-1 (Decalf et al., unpublished results). The signal inducing the early ISG wave preceded reverse transcription but required viral fusion. Virus-like particles devoid of their genome, but capable of fusing, elicited a similar ISG response, indicating that viral nucleic acids were not implicated is this sensing step (67). Importantly, this early and transient ISG induction alone conferred partial protection to macrophages against subsequent HIV-1 infection. Different viruses carrying different envelopes and thus entering MDM although different receptors exhibited similar capacities to induce this response (67). The actual sensor of viral entry involved in this process remains to be identified. Membrane perturbations, such as fusion events, can elicit antiviral responses in macrophages, *via* the stimulator of IFN genes (STING)/tank-binding kinase-1 (TBK-1)/interferon-responsive factor-3 (IRF-3) pathway (68, 69). Thus, it is tempting to consider that the plasma membrane of the macrophage represents its first line of defense, and that sensing membrane perturbations, like viral entry, as soon as it occurs would be advantageous for the rapidity of the establishment of the antiviral response (Figure 1).

Retro-transcription represents a very specific and mandatory step for retroviral replication and is the target of SAM domain- and HD domain-containing protein 1 (SAMHD1), a major restriction factor for HIV-1 replication in macrophages and other cells of the myeloid lineage, such as DCs (71, 72). Upon fusion of the HIV envelope with the host cell membrane, the viral capsid (CA) is released into the cytoplasm and the viral reverse transcriptase initiates reverse transcription of the viral RNA genome. SAMHD1 restricts HIV infection by depleting the cytosolic pool of dNTPs available for reverse transcription, *via* its deoxynucleoside triphosphate triphosphohydrolase activity (73) and possibly also by directly attacking viral RNA *via* its ribonuclease activity (74). While HIV-1 has no known factor to counteract SAMHD1 restriction, HIV-2 and several SIV strains encode the accessory protein Vpx that targets SAMHD1 for degradation (71, 72). Yet, HIV-1 is capable of replicating in macrophages, suggesting alternative mechanisms to bypass restriction.

Cyclin-dependent kinases (CDKs) phosphorylate SAMHD1 in proliferating cells, halting its activity (75, 76). Interestingly, primary macrophages in culture spontaneously and temporarily enter a G1-like state, without progressing to actual cell division, leading to CDK1 induction that limits the levels of SAMHD1 in its active form (77). This provides HIV-1 with a window of opportunity, as the virus preferentially infects these G1-like phase macrophages (77). Importantly, microglial and peritoneal macrophages recovered from mouse tissues similarly exhibit spontaneous cycling between G0 and the G1 state, suggesting a relevance for this mechanism *in vivo* (77). Other recent studies reported elevated levels of different members of the cyclin family in macrophages, rendering them permissive to HIV-1 infection, further supporting an important role for cell cycle proteins in the mechanism through which HIV-1 bypasses SAMDH1 restriction (78, 79).

Packaging Vpx into HIV-1 virions leads to a strong increase in infection efficiency of macrophages and DCs (80). However, such increased infectivity comes at the cost of detection of the viral cDNA by the cytoplasmic DNA sensor cGAS and induction of antiviral type I IFN (63). This suggests that HIV-1, unlike HIV-2, adopts a strategy of co-habitation with SAMHD1 in myeloid cells to avoid triggering antiviral immunity (81).

There is, however, a second phase of ISG induction in macrophages, peaking around 96 h postinfection that requires retro-transcription (67) and viral integration (70). This response is induced by detection of newly transcribed viral RNA by the RNA sensor retinoic acid-inducible gene I, and it requires the activity of the trans-activating (tat) HIV-1 accessory protein, responsible for the elongation of HIV-1 transcripts (70). Together, the two sensing steps confer an ISG signature in HIV-1-infected macrophages that may contribute to maintaining a low level of viral replication in this cell type (67, 70) (Figure 1).

Once viral proteins are produced, a key step in the assembly of new viral particles is the incorporation of the viral envelope. Two recently identified restriction factors, active in macrophages, target the viral envelope and thus reduce infectivity. Membrane-associated RING-CH 8 is highly expressed in myeloid cells where it retains the viral envelope glycoproteins intracellularly hence impairing their incorporation into the budding viral particles (82). The guanylate binding protein-5 (GBP5) is highly inducible by type I IFN and interferes with processing, trimming and incorporation of the HIV-1 envelope, rendering the produced virions less infectious (83). Interestingly, the viral genes encoding Env and the HIV-1 accessory protein Vpu are expressed from the same bicistronic RNA (84). Deletion of *Vpu* from the viral genome enhances Env expression and renders HIV-1 less susceptible to GBP5 restriction (83). These observations may explain the high frequencies of defective Vpu gene observed in M-tropic HIV-1 strains (85).

Not surprisingly, restriction also takes place during the late phase of HIV replication cycle in macrophages. Tetherin (or BST2) is an interferon-inducible transmembrane protein that restricts HIV-1 particle release by inserting its C-terminal end into the viral lipid bilayer (86). As a result, newly formed virions are unable to leave the surface of infected T cells; i.e., they stay tethered (87). Tetherin activity is counteracted by Vpu that mediates its surface downregulation and degradation (88). HIV-1 infection of macrophages upregulates tetherin in an apparently IFN-independent but Nef-dependent manner (89). However, despite the presence of Vpu, HIV-1-infected macrophages still express detectable levels of tetherin that appears to partially restrict viral particle release (89).

The interferon-induced transmembrane (IFITM) proteins belong to a small family of highly related proteins and act has as broad restriction factors able to interfere with the replication of many viruses. These relatively short proteins (around 130 aa) were initially characterized for their capacity to protect IFITM expressing cells from HIV-1 infection (90, 91). In addition, viral particles produced by IFITM expressing cells exhibit a reduced infectivity due to their incorporation of IFITM into the viral envelope (92). Whether present in the membrane of the target cell or the viral particles, IFITM proteins appear to impair viral fusion. Of note, endogenous IFITM3 also inhibits cell-to-cell transmission, and its inhibitory effect is stronger when it is present in viral particles than when part of the target cell membrane (92). The mechanism of action of IFITM is still unclear but probably relies on their capacity to reduce the fluidity of the membranes where they insert, thereby preventing viral fusion (or hemifusion). The precise topology of IFITM proteins is probably key to understand how they work, but this aspect is still debated. Mutagenesis studies combined with secondary structure predictions indicate that IFITM3 is a type 2 transmembrane protein that possesses an amphipathic helix adjacent to two palmitoylated cysteines (93). Silencing of IFITM1, 2, and 3 in HIV-1 producing cells resulted in increased infectivity of the viruses released (94). Importantly, the impact of such silencing was more striking in MDM than in any other cell type (94), raising the possibility that IFITM expression induced early in HIV-1-exposed MDM, as we documented (67), reduces the infectivity of the viral progeny.

In conclusion, low-level sensing of HIV-1 by macrophages results in the induction of a panoply of restriction factors that severely restricts infection. This antiviral state still allows for a certain degree of replication-competent viral production and may explain why HIV-1-infected macrophages are so resistant to the cytopathic effects that the virus exhibits in other cell types. The net effect, however, is that macrophages produce HIV-1 for extended periods of time which may favor long-term viral persistence.

## Cell Biology of HIV Assembly in Macrophages: From GAG Synthesis to Particle Release

**Figure 2:**
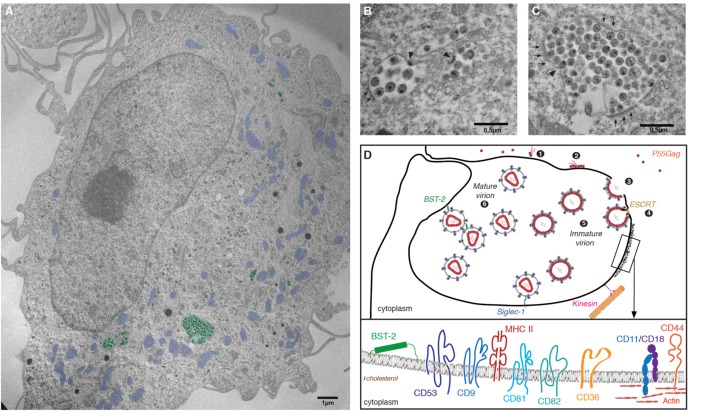
The virus-containing compartment (VCC) in macrophages. **(A)** Electron micrograph depicting HIV-1-infected monocyte-derived macrophages (MDMs). MDMs were differentiated from monocytes purified from the peripheral blood of healthy human donors, by culture over 7 days in the presence of M-cerebrospinal fluid. Cells were then infected with HIV-1 NL-AD8 and fixed and embedded in epon 5 days postinfection. Ultrathin sections were processed for electron microscopy and imaged using a Philips 120 keV. For clarity, VCC is pseudo-colored in green and mitochondria in blue. **(B,C)** Magnifications of VCC present in the MDM depicted in panel **(A)**. Arrowheads indicate viral buds. A thick molecular coat, electron dense and often associated with the VCC limiting membrane of the VCC can be seen in panel **(C)**, see arrowheads. **(D)** Schematic representation of the late phases of the HIV life cycle in macrophages. (1) Gag monomers initiate oligomerization in the cytoplasm, forming dimers with the viral genomic RNA and (2) subsequently bind the plasma membrane *via* interactions with acidic phospholipids. (3) In macrophages, high-order Gag multimerization and formation of a viral bud only occurs at the limiting membrane of the VCC. (4) Gag subsequently recruits the components of the ESCRT complex that ensure fission of the budding viral particle into the lumen of the VCC. (5) Immature viral particles accumulate inside the VCC and (6) convert into mature viral particles *via* the activity of the viral protease. The restriction factor BST2/tetherin and Siglec-1/CD169 may contribute to the retention of viral particles within the lumen of the VCC. The limiting membrane of the VCC is tightly associated with the microtubule network on which kinesins may drive the transport of the VCC toward the cell periphery. *Inset*: Magnification of a region associated with a molecular coat: the VCC limiting membrane constitutes a platform for viral assembly where Gag oligomerization will lead to virus production. Therefore, the viral and VCC membranes share similar composition. They are enriched in particular lipids such as cholesterol and transmembrane proteins. These include the tetraspanins CD9, CD53, CD81, and CD82, the scavenger receptor CD36, the integrins CD18/CD11, or the surface glycoprotein CD44 (see text for details and references). The electron micrograph depicted in this figure panel results from original work performed in our lab and has not been published elsewhere previously.

In CD4^+^ T cells and model cell lines, HIV assembly and budding take place at the plasma membrane. By contrast, in infected macrophages HIV buds into an apparently intracellular compartment, known as the virus-containing compartment (VCC). The VCC is a unique compartment with topological and biochemical properties distinct from late endosomes or multivesicular bodies, see Ref. (95). Indeed, the VCC (i) lacks classical markers of endosomal and lysosomal compartments (96, 97); (ii) possesses a near neutral pH (97); and (iii) is connected to the extracellular milieu making its lumen accessible to small membrane-impermeable dies (96, 98–100). The compartment consists of a complex membranous system of interconnected tubules and vesicles enclosing immature and mature viral particles in its lumen as well as budding virions in its limiting membrane (99) (Figure 2). The limiting membrane of the VCC possesses specific biophysical properties due in part to the particular membrane topology of the proteins that are inserted and the presence of high levels of cholesterol (101).

Evidence supports the notion that the VCC originates from intracellular sequestration of domains of the plasma membrane with a specific protein and lipid composition (102, 103). Cell surface proteins found in the limiting membrane of the VCC include the tetraspanins CD9, CD53, CD81, and CD82 (96, 104) that provide membrane rigidity; the scavenger receptor CD36 (105) that contains two cytoplasmic tails; CD44 (98), a receptor with promiscuous capacity to bind numerous ligands; and MHC II complexes (106) (Figure 2). The restriction factors IFITM, with their peculiar topology of membrane insertion, are incorporated into viral membranes, especially in viruses produced by MDM (94), and may therefore also associate with the limiting membrane of the VCC, potentially contributing to a decreased membrane fluidity. As in T cells, the ESCRT complex is in charge of promoting the fission of nascent particles (107) and ESCRT III proteins that are key players in the late abscission process have been found at the VCC limiting membrane (108). Further supporting a surface origin for the VCC, fluorescence recovery after photobleaching (FRAP) experiments demonstrated that the VCC limiting membrane and the plasma membrane are in rapid equilibrium (102).

Given that newly formed particles bud away from the cytosol toward the lumen of the VCC, they become wrapped with VCC-derived membrane (Figure 2). The proteomic analysis of purified HIV-1 particles released by infected MDM published more than 10 years ago (109) thus reflects the protein composition of the VCC limiting membrane, and still represents a valuable source of information. Studies published thereafter indeed confirmed not only the presence of the given proteins at the VCC limiting membrane but also revealed their role in the viral assembly process [see, for instance, CD36 (105), IFITM (94), and CD18 or Filamin A (110)].

An issue debated since the initial characterization of the VCC is whether some degree of viral budding can also occur at the surface of the macrophage. Indeed, some studies claimed that budding at the surface occurs alongside budding at the VCC (99, 111). A recent study provided an exhaustive examination of the site of virion assembly in individual macrophages by using a viral mutant arrested at the budding stage (103). Although such arrested buds could potentially move toward the plasma membrane, the analysis indicated that only about 5–12% of budding events occur at the cell surface. Moreover, these events were concentrated in the region where the VCC connected with the surface (103). These observations confirm that the VCC is the primary site of HIV assembly in macrophages, while budding at the surface is, at best, rare.

Thus, the specific lipid and protein composition of the limiting membrane of the VCC seem to constitute an assembly platform (101, 112), and presumably possess specific properties required for assembly and budding. However, which cellular and viral determinants direct HIV assembly and budding in macrophages to the VCC, remain poorly elucidated. The Gag polyprotein precursor drives the multistep process of HIV assembly and coordinates the activity of the cellular players involved. Recent studies indicate that viral genomic RNA is an important player/cofactor in the viral assembly process. Using elegant cross-linking immunoprecipitation sequencing coupled to membrane flotations, Kutluay et al. established that assembly starts in the cytoplasm where monomers and dimers of newly synthesized Gag bound to viral genome accumulate (113, 114). Oligomerization of Gag requires interactions with membrane enriched in cholesterol (115), *via* its MA domain that directly binds to membrane phosphatidylinositol 4,5-bisphosphate (PI(4,5)P2) (116) [see Ref. (112)]. The levels of PI(4,5)P2 in the VCC are similar to those at the surface (102) and hence should not be a determining factor that directs viral assembly to the VCC. Assembly of the viral particle at the membrane is coordinated *via* lateral interactions promoted by the CA and nucleocapsid (NC) domains of Gag, leading to a nascent virion that buds off the membrane (117). Gag possesses two RNA binding domains (NC and MA) with different specificities (115). In addition, the RNA binding specificities for the viral genome versus cellular RNA, i.e., mRNA and tRNA, present in the cytosol change during these processes finally promoting packaging of viral genomic RNA into the nascent viral particles (115).

Aiming to elucidate the role of the viral factors dictating the assembly site of HIV in macrophages, Inlora and colleagues evaluated the impact of targeted deletions or mutations in the different Gag domains, on virion assembly at the VCC (118). Viral mutants unable of high-order multimerization, due to NC substitutions, distributed equally between the VCC and the cell surface (118). This indicates that Gag initially binds the membrane arbitrarily at the surface membrane or the VCC and initiates low-order multimerization. However, in macrophages, high-order multimerization and complete virion assembly occur at the VCC but not at the surface.

Assembly of the polyprotein Gag precursor leads to the formation of a bud head and stalk. Gag further coordinates the recruitment of important cellular factors. These include the ESCRT proteins; key players critically required for proper abscission of the nascent viral particles. Gag recruits components of the ESCRT complex through its late p6 C-ter domain, thus promoting severance of the viral stalk and particle release in the VCC. The precise molecular mechanisms involved in membrane abscission have stimulated numerous studies and corresponding excellent reviews [see, for instance, Ref. (107, 119)], and thus will not be discussed here.

The molecular players implicated in the establishment and maintenance of the VCC’s intricate architecture remain poorly characterized. The cytoskeleton appears to play a key role in maintaining the integrity of the VCC. Indeed, a meshwork of filamentous actin surrounds the VCC and treating MDMs with actin-depolymerizing agents causes dispersion of the VCC throughout the cell (102). Moreover, the VCC limiting membrane is often surrounded by an electron dense and thick molecular coat visible by EM and containing CD18, a β2 integrin and its associated α integrins CD11b and CD11c (Figure 2) (120). Proteins known to interact with integrins are also associated with the molecular coat, including actin and focal adhesion scaffold/linker proteins such as talin, vinculin, and paxillin (120). These adherent complexes appear to be involved in the maintenance of the architecture of the VCC; however, CD18 silencing does not affect the amount of virus released nor its infectivity (120). Links between VCC and the autophagy machinery in HIV-1-infected macrophages have been investigated in a few studies that should be extended in the future (121).

The VCC limiting membrane appears tightly associated with the microtubule network and disruption of this network by nocodazole exposure leads to relocalization of the VCC into the perinuclear area (122). This suggested that kinesins, molecular motors associated with microtubules, could be involved in maintaining the correct positioning of the compartment. The kinesin II, KIF3A, is closely associated with VCCs in infected macrophages (122). Moreover, time-lapse microscopy in primary MDM showed paired movements of Gag and KIF3A. Importantly, KIF3A silencing in HIV-1-infected macrophages reduced viral particle release and increased intracellular Gag and the VCC volume. Overall, KIF3A is likely involved in the transport of the VCC along microtubules and toward the macrophage periphery or provides a force for particle release from the VCC (122). Other molecular motors are likely involved in the transport and positioning of the VCC but also in transport steps of viral components toward the assembly sites, i.e., the VCC (123). Future work will aim to establish at the molecular level how viral release from VCC is regulated.

While the VCC is defined by its viral content, the question of its induction by HIV or its existence in macrophages before infection was of interest. Analysis of primary MDMs by ultrastructural approaches suggested that indeed similar compartments are present in non-infected macrophages (96, 99, 120). Confocal microscopy confirmed the presence in non-infected MDMs of compartments sharing specific features with the VCC; i.e., containing CD36 and CD9, rapid accessibility to small dextran and therefore connected to the external medium (105). The direct proof of HIV capacity to highjack such compartments was obtained when transduced macrophages exhibiting CD36-GFP^+^ compartments were subjected to time-lapse epifluorescent microscopy after infection with HIV-1 Gag-iCherry allowing the visualization in real time of Gag recruitment and accumulation only to preexisting CD36^+^ compartments (105). Thus, in macrophages, HIV-1 hijacks these CD36^+^ compartments for viral assembly (105), and expands them (96).

Viral particles accumulate in the VCC over time (100). Infected MDMs initially contain sparse, barely filled VCCs, but as the time after infection progresses the compartments become crowded with virions and Brownian-like movements of particles within the VCC become highly limited as shown by FRAP experiments (100). In parallel, viral release into the extracellular media decreases over time, suggesting that viral particles remain within the VCC (100). The restriction factor tetherin is concentrated in the VCC where it may connect viral particles to each other or to the VCC limiting membrane, hence apparently tethering virions to the compartment (89, 124). Indeed, silencing tetherin expression increases viral release and reduces the size of the compartment (89).

Whether HIV-1 release from the VCC is inducible remains indeed elusive. The connections between the VCC and the membrane appear too narrow to allow passive viral diffusion to the extracellular media (99). The release of HIV-1 from infected MDMs can be induced by exposure to extracellular ATP, *via* activation of the P2X7 purinergic receptor that triggers a drastic remodeling of the cytoskeleton and the VCC, accompanied by sudden release of the viral particles packed within the VCC (125). However, we also propose that the highly dynamic nature of the plasma membrane of macrophages, which is subjected to a very active flux of exocytosis and endocytosis/phagocytosis, may promote the temporary widening of the VCC connections to the plasma membrane and allow the release of viral particles to the extracellular media. Supporting this hypothesis, antibodies specific for VCC components, such as tetraspanins (96), gp120 (126), or CD36 (105) had access to the VCC only after several hours of incubation at 37°C and not at 4°C. These observations suggest that the access of extracellular molecules to the VCC depends on the dynamics of the macrophage’s plasma membrane, which possibly promotes a frequent opening of the connections between the lumen of the compartment and the surface. Future insight into the role of the cytoskeleton and its associated proteins in regulating the dynamics of the VCC and its impact on viral release may open new avenues to pharmacologically target the VCC (123, 127).

Whether tissue macrophages *in vivo* possess VCCs remains poorly investigated. Indeed, our current view of the VCC results almost exclusively from studies *in vitro* with MDMs. Of note, former ultrastructural analysis of tissue macrophages from patient’s organs confirmed the presence of mature and immature virions in intracellular compartments (128, 129). Accumulation of viral particles within VCC in macrophages *in vivo* could be advantageous for the virus to be less accessible to (i) soluble immune mediators such as neutralizing antibodies as observed *in vitro* (126), (ii) to innate sensors that are cytoplasmic or endosomal, and (iii) to anti-pathogen effector mechanisms such as reactive oxygen species or acidic pH.

Direct cell-to-cell transfer is more efficient for spreading HIV infection than the cell-free route (130). This process involves the formation of a stable interface between an infected and an uninfected cell, known as viral synapse. Interaction between the viral envelope present at the membrane of the infected cell and CD4^+^ in the target cell initiates formation of the viral synapse, which is then maintained *via* additional interactions between cell adhesion molecules (131). Directed release leads to virion accumulation at the viral synapse and efficient infection of the target cell (132). Macrophages can transfer HIV directly to T cells (133), and the VCC appears to play a role in this process. Real-time imaging suggested a recruitment of the compartment to the proximity of viral synapses leading to subsequent T cell infection (134, 135), and the VCC markers CD9, CD18, and CD81 were found enriched at the macrophage to T cell interface (124). However, the precise mechanisms underlying viral transfer from infected macrophages to target cells (T cells or macrophages) remains incompletely understood as compared with T cell to T cell transfer and thus deserve further studies.

Despite their monocytic origin MDDCs are rather resistant to HIV-1 infection as compared with MDMs. Yet, MDDCs, when activated by LPS, can capture and retain vial particles in surface-connected compartments that bear some resemblance with the VCC. Although activated MDDCs are resistant to the infection and do not produce new viral progeny, they can efficiently transfer captured viruses to activated CD4^+^ T cells that get then productively infected. This transfer mode known as infection *in trans* [see accompanying review by Izquierdo-Useros or Izquierdo-Useros et al. (136)] appears to be highly related to the virological synapse established between HIV-1-infected T cells and target cells (137). This process is thought to play an important role in viral dissemination at the early stages of HIV-1 infection at mucosal entry sites (138). Intravital microscopy revealed that subcapsular sinus macrophages from the peripheral lymph nodes can capture and transfer HIV-1 to target cells without getting productively infected in the process (139). Viral capture is mediated by the sialoadhesin CD169/SIGLEC1 that binds gangliosides embedded in the envelope glycoprotein (139). Interestingly, CD169-mediated capture of HIV-1 in macrophages leads to virion retention in the VCC and subsequent transfer to and productive infection of CD4^+^ T cells (140). Remarkably, viral particles captured *via* CD169 intermingled with virions endogenously produced by the macrophage in the same VCCs (140), suggesting that the VCC is not only the site of HIV budding and assembly in macrophages but also a compartment of retention of particles captured from the extracellular media. Indeed, compartments with topologies resembling the VCC have been described in the past after exposure of macrophages to various particulate matter, including latex, cholesterol, or low-density lipoprotein (95).

## Macrophages and the HIV Reservoir in the Post-Art Era

While ART can efficiently prevent AIDS by restoring CD4^+^ T cell counts and suppressing viral load to undetectable levels, it fails to provide a sterilizing cure (141). A viral reservoir remains stable in HIV-infected patients under prolonged therapy (142, 143), and is responsible for the quick viral rebound observed within weeks after ART interruption (144). The cumulative toxicity and the cost associated with lifelong ART made it imperative to devise new strategies to eliminate or curb the viral reservoir (145). Unfortunately, such goal has remained elusive.

At the cellular level, the HIV reservoir during ART is mainly composed of resting memory CD4^+^ T cells that are latently infected; i.e., cells bearing integrated, transcriptionally silent, but replication-competent proviruses (146). The mechanism behind HIV latency is not fully understood but likely results from multiple factors acting together, such as sequestration of cellular transcription factors in the cytoplasm, epigenetic regulation, or the action of transcriptional repressors [reviewed in Ref. (147)]. T cells with central memory (T_CM_), transitional memory (T_TM_), and effector memory (T_EM_) phenotypes contain the highest levels of latent HIV-1 (148–150). A major breakthrough in the characterization of the latent CD4^+^ reservoir is the recent identification of CD32a as a surface marker highly enriched in circulating cells harboring replication-competent quiescent proviruses (151). The HIV reservoir is seeded very early after infection (within 2–3 days) (152, 153), and its size remains stable even after years of suppressive therapy (154, 155).

Whether residual viral replication under ART, the so-called active reservoir, contributes to HIV persistence is a rather contentious issue in the field (156). Low-level viremia (“Blips”) can be detected in patients under therapy (157, 158). However, HIV shows little sign of genetic evolution during ART (159, 160), and the emergence of drug-resistant virus is remarkably low (158, 161), suggesting that ongoing residual replication does not significantly influence long-term viral persistence. However, a recent high-depth temporal analysis of the phylogeny of viral sequences from the blood and lymph node of patients under ART revealed a constant replenishment of the circulating reservoir as a result of low-level viral replication in sanctuary sites within lymphoid tissues (162). Experimental evidence for the existence of such lymphoid sanctuaries has emerged in the last years. B cell follicles and more specifically germinal centers have long been known as primary sites of HIV replication (163), possibly due to lower antiretroviral drug penetration (164), exclusion of cytotoxic CD8^+^ T cells (165, 166), and retention of infectious virions within immune complexes on the surface of follicular dendritic cells (167). In patients under treatment and with an aviremic status, CD4^+^ T cells with a T follicular helper phenotype that reside within germinal centers are the major source of residual infectious virus and contain the highest levels of HIV DNA (168–170).

These recent advances highlight the importance of accurately defining the HIV reservoir, with respect to its cellular and anatomic composition (171). However, studies evaluating the importance of cellular reservoirs other than CD4^+^ T lymphocytes have been scarce and, for the most part, non-conclusive. Analysis of the rebounding viral sequences in patients after therapy interruption revealed that they differ from proviral sequences integrated in resting CD4 T cells (172). Recovery of M-tropic sequences among the pool of rebounding virus has been recently reported (173). However, there is no readily available method to measure viral rebound from macrophages or other cellular reservoirs, equivalent to the viral outgrowth assay (VOA) typically used to quantify the latent CD4^+^ reservoir (171). Usually, VOAs require the culture of several million purified cells for accurate quantification of the CD4 reservoir, due to the rarity of latently infected cells (150), making an adaptation of the assay to tissue macrophages unfeasible. Alternatively, quantification of viral nucleic acids provides a more practical manner to evaluate the macrophage reservoir (174). In patients under ART, HIV DNA and/or RNA has been detected in alveolar (175) and duodenal (41) macrophages, microglia in the brain (39), as well as in liver Kupffer cells (176).

Evaluation of the HIV reservoir based on cell-associated DNA or RNA tends to largely overestimate the pool of replication-competent virus, as many defective viral genomes accumulate in patients (177, 178). Also, macrophages may acquire viral DNA *via* phagocytosis of infected T cells (51), as discussed previously. In the absence of a reliable outgrowth assay to measure the macrophage HIV reservoir from treated patients, animal models become a valuable alternative.

In a recent study with Asian macaques chronically infected with SIV, replication-competent virus could be recovered from macrophages purified from the spleen and mesenteric lymph nodes, using a modified version of the VOA (43). However, in macaques that had been under ART for 5 months, no replication-competent virus could be recovered after macrophage culture, from any of the animals under study and despite the presence of detectable SIV-DNA in macrophages in 40% of the animals (43). A possible limitation of this study is the small number of macrophages that could be purified for the viral outgrowth experiments. However, in parallel experiments, replicating virus could be recovered after culture of purified memory CD4^+^ T cells from all animals under therapy (43). This suggests that, in the SIV model, the macrophage reservoir, if existent, is clearly smaller than the memory CD4 reservoir.

T cell-deficient humanized mice can be generated by transferring CD34^+^ human hematopoietic cells into NOD/SCID mice. These mice are reconstituted with human myeloid cells and B cells, but completely devoid of T cells (179). As only macrophages can sustain HIV replication, these myeloid-only mice (MoM) provide a valuable model to test the HIV macrophage reservoir without the confounding effects conferred by the presence of the more abundant CD4^+^ T cell compartment. When infected with macrophage-tropic HIV-1, MoM present sustained viremia, and viral nucleic acids were detected in macrophages of the liver, bone marrow, spleen, lungs, and brain (179). Initiation of ART in infected MoM leads to undetectable viremia within 2 weeks, and a drastic reduction in the levels of HIV DNA and RNA in the tissues. Importantly, ART interruption led to viral rebound about 7 weeks later, although only in three out of nine animals under study (180). This represents a significantly longer period of viral remission after treatment interruption when compared with T cell-sufficient humanized mice, where rebound occurs within 1–2 weeks after ART interruption (181). Due to the short life span of the MoM mice, the authors could not extend the study and evaluate whether the non-rebounding group of mice would eventually show signs of reactivation of the infection (180).

The absence of T cells in the MoM model of HIV infection, certainly constitute a large deviation from the regular course of HIV infection in humans. Nevertheless, these observations provide the first direct evidence for HIV persistence in macrophages, in the setting of suppressive therapy. Whether such persistence is due to latent infection or ongoing residual replication remains unclear from the data available, as both viral DNA and RNA dropped to undetectable levels in most treated mice (180). Efforts to purge the CD4^+^ T cell reservoir have mostly employed the “shock and kill” approach (145); a latency-reversal agent (LRA) is initially administered to reactivate viral production in latently infected cells (shock), and followed by an immune-modulatory intervention that renders infected cells susceptible to destruction by the immune system (kill) (182). However, HIV latency in macrophages is not well understood (183), and LRAs that efficiently purge the CD4 reservoir may not have a similar effect in latently infected macrophages. Worse, if the macrophage reservoir is maintained mostly *via* residual ongoing replication or retention of infectious viral particles within VCCs then LRA therapy will be of negligible effect.

Definitive proof of the macrophage reservoir will require demonstration in human patients under therapy. This will likely require more sensitive methods of measuring the reservoir. A promising alternative is the recent report of an *in vivo* VOA, wherein cells from patients with undetectable viremia are transplanted into humanized mice (184). This method appears more sensitive than *in vitro* VOAs, although its widespread application will likely be hampered by the costs associated (185).

## Macrophages During Chronic Disease in Treated HIV-1 Infection

Introduction of ART effectively halted the AIDS pandemic, improved health, and prolonged the life of patients. However, a new group of problems, commonly known as “non-AIDS-related conditions,” is emerging in HIV patients with long-term suppressed viremia (186). People living with HIV are at increased risk of developing, among others, cardiovascular and neurocognitive disease, osteoporosis, or cancer (187).

Persistent inflammation appears to lie at the origin of these pathologies, although its causes remain incompletely elucidated and may involve multiple factors. Microbial translocation across the gut mucosa is a well-established cause of systemic inflammation during HIV-1 (188, 189). Long-term suppressive therapy does not completely reconstitute the pool of CD4^+^ T cells in the gut mucosa, particularly those of the Th17 subset (190). This leads to loss of integrity of the epithelial mucosa and translocation of bacterial products through the lamina propria to mesenteric lymph nodes and extranodal sites (189). These microbial products engage pattern-recognition receptors in cells of the innate immune system, particularly monocytes, macrophages and DCs, leading to widespread production of inflammatory mediators (191). Indeed, myeloid cell-derived biomarkers of microbial translocation, such as IL-6, soluble CD14 (sCD14) or sCD163 are found elevated in ART-treated individuals, as compared with age-matched controls, and are strongly associated with premature mortality of HIV-infected individuals (191, 192). This persistent pro-inflammatory state appears to feedback on intestinal macrophages as they become unable to phagocyte microbial debris in the lamina propria and are thus unable to halt this inflammatory cycle (193). Importantly, persistent systemic inflammation drives the occurrence of non-AIDS comorbidities. For instance, inflammatory monocytes migrate to the heart and contribute to HIV-associated myocarditis (194).

Before the implementation of ART, more than half of HIV-infected patients exhibited HIV-1-associated dementia (HAD); a broad term used to described symptoms of cognitive impairment, including psychiatric disorders, loss of motor coordination, and in severe cases, HIV-1-associated encephalitis (195). The incidence of HAD has dramatically decreased with ART, but a set of milder cognitive problems have emerged and are collectively known as HIV-associated neurological disorders (HAND) that still affect about half of the HIV-1-infected population (196).

Residual viral replication in the CNS and neuronal death has been proposed has an explanation for the occurrence of HAND (197). This effect is probably indirect, as neurons and cells of the macroglia do not support productive HIV infection. Instead, residual viral replication in macrophages leads to the production of inflammatory mediators with neurotoxic action (196). While it is challenging to assess productive infection of brain-resident cells, macrophages are known to contain the highest levels of viral nucleic acids of all CNS cell populations (198). Both perivascular macrophages and microglial cells are targeted by HIV in the CNS (199) and HIV DNA has been detected in brain macrophages from patients under long-term therapy (39). HIV RNA persists in the cerebrospinal fluid (CSF) of ART-treated patients even after suppression of plasma viral RNA to undetectable levels (200), and genetic analysis revealed a significant compartmentalization between the CSF and plasma viral populations (201). Importantly, viruses isolated from the CSF are frequently M-tropic (202). Taken together, these studies suggest that the CNS is a tissue reservoir of HIV-1 during ART, likely maintained through low-level viral replication in resident macrophages (203).

Systemic inflammation is also likely to play a role in the progression of HAND. There is a strong association between circulating levels of sCD14 and the development of neurological disorders in HIV-1 infected individuals (204). It has been proposed that microbial products activate circulating monocytes, particularly those of the CD16^+^ subset that subsequently cross the blood–brain barrier and differentiate into a pro-inflammatory macrophage population in the brain by producing chemokines, cytokines, and neurotoxic factors such as nitric oxide (48). Supporting this model, CD14^+^CD16^+^ cells accumulate in the white matter and perivascular space of brains from non-treated patients (205). Also, these CD14^+^CD16^+^ monocytes are capable of transmigrating across *in vitro* models of the blood–brain barrier in response to the chemokine CCL2 (206).

These selected examples highlight the pathological role played by macrophages and other myeloid cells in non-AIDS conditions that afflict HIV-1-infected patients with suppressed viremia. It is thus crucial to devise new therapies able to complement ART and capable of targeting the persistent inflammation that drives these morbidities.

## Conclusion

Because of their localization in many tissues, their long life span and the unique nature of their interaction with HIV-1, macrophages play a key role in HIV-1 pathogenesis. The last decade of research brought new and exciting insight into the ontogeny and functional specialization of tissue-resident macrophages. Whether these recently described macrophage properties, such as their proliferative potential, are explored HIV for dissemination and persistence remains unknown, but this subject should deserve increased interest in future studies. Macrophages are susceptible to HIV-1 infection but also sense the virus and thus participate in the general immune activation observed in infected patients. However, initiation of the antiviral immune response relies on the DC population whose capacity to perform antigen presentation and deal with infection is highly organized in space and time. This dual function appears to be ensured by a division of labor between DC subsets (207). While cDC1 (CD141^+^) are resistant to productive HIV-1 infection, they can cross-present viral antigens derived from cDC2 (CD1c^+^) that are susceptible to HIV-1. Thus, this dissociation of the viral infection and the antigen presentation function provides to DC populations the capacity to elicit antiviral immune responses and prime T cell responses (207). How macrophages cross talk with DCs and contribute to the antiviral response remains obscure. Reciprocally, how HIV succeeds to cope with these immune cells specialized in antiviral immunity is similarly incompletely understood. Future work addressing these questions should keep on producing exciting results enlightening our general comprehension of the virus–immune system relationships.

## Author Contributions

VR and PB wrote the review. MS-R performed the electron microscopy. NR, PB, and VR designed the cartoon. All the authors edited the review.

## Conflict of Interest Statement

The authors declare that the research was conducted in the absence of any commercial or financial relationships that could be construed as a potential conflict of interest.
